# Thermosensitive Chitosan/Gelatin Hydrogels in Traditional Chinese Veterinary Medicine: A Prospective Review on Modernizing Acupoint Embedding

**DOI:** 10.3390/gels12030193

**Published:** 2026-02-26

**Authors:** Yingying Xie, Xuequan Hu, Ying Li, Jianfa Wang, Rui Wu

**Affiliations:** 1 College of Animal Science and Veterinary Medicine, Heilongjiang Bayi Agricultural University, Daqing 163319, China; 2 College of Biology and Agriculture, Jiamusi University, Jiamusi 154007, China

**Keywords:** chitosan/gelatin hydrogels, thermosensitive hydrogels, Traditional Chinese Veterinary Medicine (TCVM), acupoint embedding therapy, drug delivery

## Abstract

Thermosensitive hydrogels have emerged as promising intelligent biomaterials for minimally invasive delivery and targeted therapy. Chitosan/gelatin thermosensitive hydrogels, integrating the biocompatibility, biodegradability, and antibacterial activity of chitosan with the excellent adhesive properties of gelatin, exhibit unique injectability, temperature-responsive gelation, and tunable physicochemical properties. This review systematically summarizes the key performance parameters of chitosan/gelatin thermosensitive hydrogels, including injectability, gelation characteristics (with sol-gel transition tunable between 37 and 42 °C to match diverse species’ body temperatures), mechanical properties, biocompatibility, degradation behavior (tunable from 1 to 8 weeks), drug-loading/release capabilities, and multi-stimuli responsiveness (pH/ROS/enzyme). It focuses on exploring their feasibility and suitability as acupoint embedding materials in Traditional Chinese Veterinary Medicine (TCVM), addressing the technical bottlenecks of traditional acupoint catgut embedding (e.g., unstable degradation, insufficient biocompatibility, and lack of drug-loading capacity). While recent studies have demonstrated the utility of such hydrogels in human disease models (e.g., rheumatoid arthritis and Parkinson’s disease), their translation to veterinary acupoint therapy remains largely unexplored. The prospective application of these hydrogels in treating common animal diseases (e.g., piglet diarrhea, canine degenerative joint disease, and equine laminitis) is, therefore, proposed and analyzed as an illustrative paradigm, emphasizing its integrated “stimulation–drug delivery” function and cross-species adaptability. Additionally, the current challenges (e.g., animal-specific formulation optimization, unclear mechanism of action, and insufficient long-term safety data) and future research directions (e.g., veterinary-specific formulation development, mechanistic exploration, and clinical translation) are highlighted. This review aims to promote the interdisciplinary integration of TCVM and smart biomaterials, provide precision strategies for animal disease treatment, and ultimately contribute to the modernization and standardization of TCVM technologies.

## 1. Introduction

### 1.1. Research Background of Chitosan/Gelatin Hydrogels

**Figure 1:**
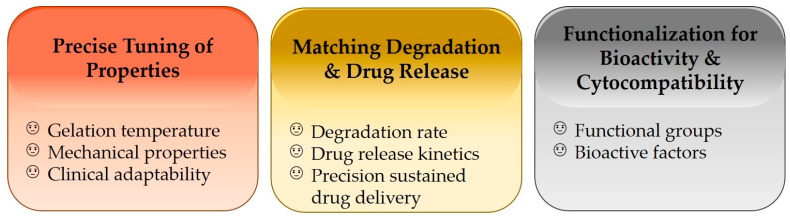
Current research background of chitosan/gelatin thermosensitive hydrogels.

As a crucial category of intelligent biomaterials, thermosensitive hydrogels enable minimally invasive delivery, targeted colonization, and long-lasting action in the biomedical field, owing to their body temperature-triggered reversible sol-gel phase transition. Chitosan, a natural cationic polysaccharide, combines biocompatibility, biodegradability, and antibacterial activity. However, single-component chitosan hydrogels exhibit inherent drawbacks, including low mechanical strength and poor tunability of gelation temperature [1]. Gelatin, a natural hydrolyzate of collagen, exhibits excellent cell adhesive properties and biocompatibility [2]. When gelatin is compounded with chitosan, a synergistic network is formed via intermolecular hydrogen bonding and electrostatic interactions, which significantly optimizes the comprehensive performance of the hydrogels [3]. Chitosan/gelatin thermosensitive hydrogels undergo a sol-gel transition at near-physiological temperatures via physical or chemical cross-linking. They demonstrate broad application prospects in fields such as drug delivery and tissue engineering [4,5]. Nevertheless, several challenges remain (Figure 1): (1) further precise tuning of gelation temperature and mechanical properties is required to meet diverse clinical needs; (2) the hydrogel degradation rate needs to be better matched with drug release kinetics for precision sustained drug delivery; and (3) introducing functional groups or compounding bioactive factors to enhance hydrogel bioactivity and cytocompatibility remains a key research direction. Notably, the intelligent properties and favorable biosafety of these hydrogels provide novel insights for their application in interdisciplinary fields integrating traditional medicine and modern materials science, such as acupoint therapy in Traditional Chinese Medicine (TCM) and Traditional Chinese Veterinary Medicine (TCVM).

### 1.2. Current Status and Challenges of Acupoint Catgut Embedding Therapy

**Figure 2:**
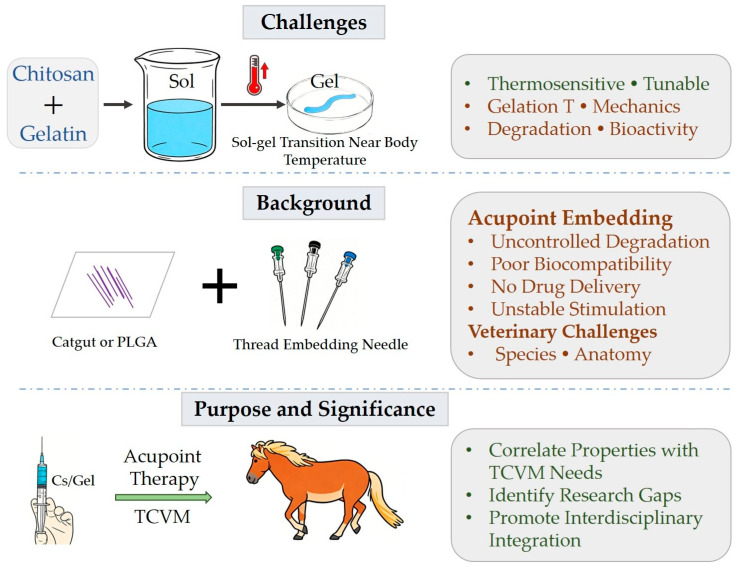
Schematic overview of the introduction: from hydrogel background to review objectives.

Acupoint therapy is a vital component of Traditional Chinese Medicine, encompassing various forms such as acupuncture, moxibustion, and acupoint catgut embedding. Acupoint catgut embedding therapy involves implanting absorbable threads into acupoints to achieve therapeutic goals through continuous stimulation of the acupoints by the thread [6]. This procedure is typically minimally invasive but still requires puncture or a small incision to insert the thread into the subcutaneous layer. Traditional acupoint embedding materials mainly include catgut and collagen sutures [7]. However, these materials face issues such as unstable degradation rates and difficulties in controlling stimulation intensities (Figure 2). In recent years, with the advancement of materials science, the application of smart materials in acupoint therapy has gradually gained attention. For instance, studies have utilized thermosensitive hydrogels for acupoint embedding, leveraging their temperature-responsive properties to achieve sustained acupoint stimulation [8]. Furthermore, nanomaterials have been employed in acupoint drug delivery systems to enhance therapeutic efficacy through the targeting and sustained-release properties of nanocarriers [9].

However, the modern application of acupoint therapy still faces numerous challenges. On one hand, the mechanism of action of acupoint therapy is not fully elucidated, and it lacks a robust scientific theoretical basis to support its clinical practice. On the other hand, the stimulation method and intensity of traditional acupoint therapy are difficult to control precisely, and the repeatability and stability of therapeutic effects need improvement. Additionally, the biocompatibility and safety of acupoint drug delivery systems require further validation to ensure their reliability in clinical application. These challenges are prevalent in both human and veterinary medicine. They are even more pronounced in veterinary clinical practice due to the diversity of animal species and anatomical/physiological differences (Figure 2). Therefore, developing a novel smart embedding material that can achieve controllable stimulation intensity, long-lasting action, and integrate safety with therapeutic function is a key requirement for promoting the modernization of acupoint therapy (including TCVM).

### 1.3. Purpose and Significance of the Review

This review systematically outlines the key performance parameters and regulatory mechanisms of chitosan/gelatin thermosensitive hydrogels, with a focus on exploring their feasibility and suitability as acupoint embedding materials in TCVM. It delves into the application potential of this material in animal disease treatment. The core significance of the review lies in (Figure 2): (1) establishing a correlation system between the performance of chitosan/gelatin hydrogels and the requirements of TCVM acupoint embedding, providing theoretical support for the veterinary-specific optimization of the material; (2) identifying the animal-specific technological gaps in existing research to provide precise guidance for subsequent research directions; and (3) promoting the interdisciplinary integration of TCVM therapies and smart biomaterials, offering new strategies for the precise treatment of animal diseases, and aiming to contribute to the long-term advancement of TCVM technology in terms of standardization, modernization, and international recognition.

## 2. Key Parameters and Performance of Chitosan/Gelatin Thermosensitive Hydrogels

**Figure 3:**
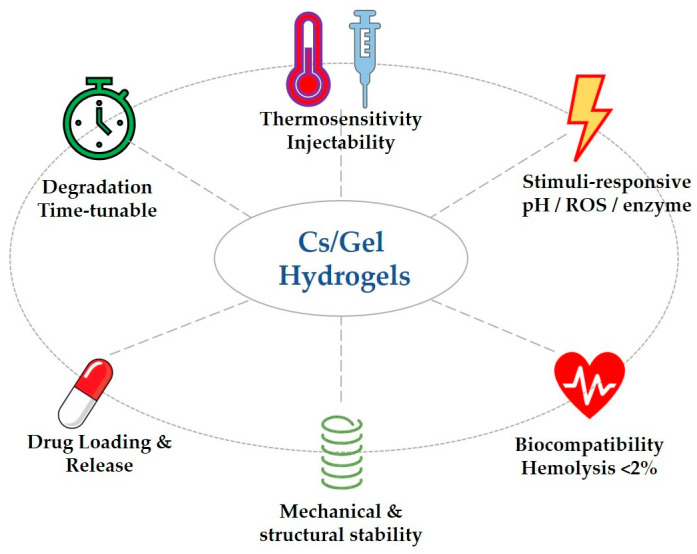
Schematic illustration of the key performance parameters of chitosan/gelatin thermosensitive hydrogels.

The performance of chitosan/gelatin thermosensitive hydrogels forms the basis of their biomedical applications, relying on the synergistic optimization of a series of physicochemical parameters and biological functions. These materials maintain an injectable sol state at room temperature and rapidly gelate upon entering the body due to temperature triggering, forming a three-dimensional network structure ideal for carrying drugs, cells, or active factors. This chapter systematically analyzes the key parameters affecting their performance, including injectability and gelation properties, mechanical properties and structural stability, biocompatibility and degradation properties, drug-loading capacity and release properties, and multi-stimuli-responsive (e.g., pH/ROS/enzyme) synergistic mechanisms (Figure 3).

### 2.1. Injectability and Gelation Properties

Injectability and gelation properties are the core prerequisites for the clinical translation of chitosan/gelatin thermosensitive hydrogels, especially in minimally invasive acupoint intervention therapy, directly determining their operational convenience and in vivo forming efficacy. Injectability is typically evaluated through viscosity, shear-thinning behavior, and injection force, while gelation properties focus on sol-gel transition temperatures, gelation time, and transition mechanisms.

In injectability evaluation, viscosity is a key indicator. The CMH2-type carboxymethyl chitosan/hyaluronic acid thermosensitive hydrogel exhibits a viscosity of 2133.4 mPa·s, showing good fluidity at room temperature. It rapidly gels within approximately 315 s when the temperature is increased to 37.8 °C (a condition mimicking human body temperature) [10]. Based on these properties, the original study suggested its potential suitability for transdermal or intracavitary injection in livestock and pets. Injection force tests of a chitosan/collagen hydrogel incorporating cuttlebone-derived nanohydroxyapatite (CB-nHA) were performed under simulated injection conditions (at 25 °C, using a 3 mL syringe with an 18 G needle at an injection rate of 0.3 mm·s^−1^). The injection force for the 5–10% CB-nHA concentration groups remained at 5.12 ± 0.60 N, with no significant increase in injection resistance, whereas the injection force for the 15% CB-nHA concentration group increased to 6.42 ± 0.22 N, exhibiting discontinuous droplet flow [11]. This result indicates that low-to-medium concentrations of inorganic nanofillers can maintain good injectability while enhancing mechanical properties, providing a basis for optimizing veterinary formulations—a hypothesis that warrants testing with species-specific anatomical parameters.

Shear-thinning behavior is a key rheological characteristic for hydrogel adaptation to fine needle injection. The quaternized chitosan/gelatin/laponite (QCS/GEL/LAP) composite hydrogel exhibits pronounced shear-thinning behavior (0.1–100 s^−1^) and rapid network recovery upon shear cessation, characteristics that are recognized as favorable for fine-needle injection [12]. This rheological profile implies potential suitability for delivering the hydrogel into deep acupoints in animals (e.g., porcine Pishu, canine Shenshu) via 21 G needles, although direct experimental confirmation in these target species and acupoint locations has not yet been reported. This rheological property effectively resolves the core contradiction between “precise delivery” and “in situ formation” in animal acupoint injection, being especially suitable for animals with smaller body size or deeper acupoints.

The regulation of gelation temperature and time must strictly match animal body temperature and clinical operation requirements. The gelation properties of chitosan/β-glycerophosphate/gelatin (CS/β-GP/Gel) hydrogels primarily depend on gelatin concentration, β-GP content, and the effects of additives: gelatin enhances gel network stability through intermolecular hydrogen bonds and electrostatic interactions, and the transition of its α-helix structure to a β-sheet conformation can optimize the hydrogels’ microstructure [13,14]. β-GP acts as a thermosensitizer, promoting temperature-dependent physical cross-linking by neutralizing the positive charge of chitosan amino groups to reduce intermolecular repulsion [15,16]. High concentrations of β-GP can accelerate gelation (gel time <5 min [17]) but may cause cytotoxicity, whereas low concentrations may lead to excessively long gelation times (e.g., the slow gelation issue in traditional CS/β-GP systems [18]). Studies show that the CS/β-GP hydrogel system can form a gel within 3–5 min under simulated physiological conditions (37 °C, pH 7.4), rendering it suitable for in vivo injection in humans [19]. For TCVM applications, the formulation must be re-optimized to match the higher body temperature of livestock and poultry (37–42 °C). Adjusting β-GP and gelatin concentrations is a promising strategy to elevate the gelation temperature and shorten gelation time, thereby aiming to achieve rapid in situ fixation at the acupoint site while maintaining cytocompatibility. This adaptation represents a key translational step that requires systematic investigation. Additionally, introducing L-lysine or L-glutamic acid can shorten gelation time (Chitosan-L-lysine/β-glycerophosphate (CS-Lys/β-GP) system) and enhance thermal stability [15], a strategy that can further optimize the cross-species adaptability of hydrogels.

### 2.2. Mechanical Properties and Structural Stability

Mechanical properties and structural stability directly determine the service life and functional performance of hydrogels within animals, requiring targeted optimization based on the acupoint microenvironments of different animals (e.g., muscle contraction areas, loose connective tissue regions). Mechanical property evaluation primarily includes compressive modulus, tensile strength, and viscoelasticity (storage modulus G′ and loss modulus G″). Structural stability is quantitatively assessed through swelling ratio, degradation rate, and microstructural characterization, ultimately achieving mechanical adaptation of the hydrogel to the acupoint microenvironment and long-term functional maintenance.

Compressive modulus is a core indicator for evaluating the load-bearing capacity of hydrogels. Pure chitosan/gelatin hydrogels exhibit weak mechanical properties; the compressive modulus of the carboxymethyl chitosan–gelatin–β-glycerophosphate composite hydrogel (CMC/G/β-GPh-12.5) is only 3.4 kPa [20]. The elastic modulus of quaternized chitosan hydrogel is even as low as 0.11–1 kPa, making it difficult to withstand dynamic mechanical stimuli such as animal muscle contraction [21]. These limitations stem from the deformability of polymer chains and the loose structure of network interstices, requiring external reinforcement strategies for improvement. Mechanical properties can be significantly enhanced through nanocomposite reinforcement strategies: in alginate/chitosan/gelatin nanocomposite hydrogel, the introduction of nano-SiO_2_ significantly optimized cross-linking density and the compressive modulus significantly increased compared to the pure system [22]. Fiber reinforcement strategies are also highly effective; after introducing modified polycaprolactone (PCL) microfibers into composite hydrogels, the compressive modulus can reach 20.35 ± 2.50 MPa, with a compressive strength of 12.32 ± 1.35 MPa [23]. Furthermore, adjusting chitosan concentration enables gradient control of mechanical properties [13]: when chitosan concentration increases from 3 wt% to 4.5 wt%, the hydrogel strength increases from 106 ± 8 kPa to 168 ± 12 kPa and the compressive modulus increases from 50 ± 9 kPa to 102 ± 14 kPa, providing a flexible solution for adapting to different acupoint mechanical environments [24].

Viscoelasticity is a core indicator for hydrogels to simulate the mechanical behavior of natural animal tissues. The storage modulus (G′) of CS/β-GP/Gel hydrogel is consistently higher than the loss modulus (G″), indicating dominant elastic behavior with good energy storage and recovery capabilities. This can reduce permanent gel deformation during animal movement [13]. Temperature sweep tests show that the melting temperature (Tm) of composite hydrogels (e.g., systems containing gelatin) is slightly higher than that of pure gelatin hydrogels, indicating improved thermal stability and the ability to maintain viscoelastic properties over a wider temperature range. This is particularly important for applications simulating body temperature environments, such as drug delivery or tissue engineering [21,25,26]. This mechanical stability is crucial for long-term embedding therapy, as it is hypothesized to enable the hydrogel to provide sustained mechanical stimulation to the acupoint during an animal’s daily activities. This hypothesized mechanism, while grounded in the well-established relationship between viscoelasticity and shape retention, has not yet been directly validated in TCVM acupoint settings and, therefore, warrants dedicated experimental investigation.

The regulation of structural stability requires achieving animal-specific matching of swelling ratio and degradation rate. The swelling ratio of chitosan/gelatin hydrogels typically ranges from 10 to 40 times, controlled by cross-linking density and component ratio: at a gelatin-to-chitosan ratio of 7:3, the hydrogel swelling performance is optimized, supporting local cell proliferation and drug release at the acupoint [27], while a 5:1 ratio system exhibits a further increased swelling ratio, suitable for treating animal wound in dry environments [28]. Cross-linker concentration also affects swelling behavior; increasing genipin concentration increases the number of cross-linking points, compresses the gel network pores, and reduces water molecule penetration resistance, thereby controlling the swelling ratio within an ideal range [29]. The regulation of degradation rate needs to precisely match the treatment cycle of animal diseases: The enzyme sensitivity of the gelatin component makes it susceptible to protease degradation, while chitosan can modulate the overall degradation rate. A gelatin/chitosan hydrogel (7:3, gelatin:chitosan) exhibited a moderate degradation rate in in vitro assays, making it suitable for short-term therapeutic applications (e.g., 1–4 weeks for piglet diarrhea) [27]. Conversely, excessive chitosan reduces mechanical strength and accelerates degradation speed [30]. Similarly, adding hyaluronic acid (HA) can increase cross-linking point density, prolonging the degradation cycle, adapting to the treatment needs of chronic diseases [31]. It should be noted, however, that the correlation between degradation kinetics and therapeutic efficacy—particularly the assumption that a prolonged degradation cycle directly translates into extended acupoint stimulation—remains inferential at this stage and has not been empirically established in veterinary acupoint models.

Microstructure is the core factor determining mechanical properties and stability. Scanning electron microscopy (SEM) observations show that the pore structure aperture of chitosan/gelatin hydrogels ranges from 50 to 200 μm. Pore size and distribution can be controlled by chitosan concentration and cross-linker dosage [32]. Decreasing pore size (e.g., from 37 μm to below 20 μm) can significantly increase the compressive modulus, due to the enhanced molecular chain entanglement and interfacial interactions in the high-density porous network [32,33]. Simultaneously, a small-pore structure (average < 200 μm) can restrict the diffusion of enzymes and water molecules, delaying biodegradation. For example, the degradation rate of multilayer hydrogels under pH 8 conditions is below 42%, significantly better than that of single-layer structures (>80%) [34]. Similarly, optimizing pore structure (e.g., via heat treatment or cross-linking) can reduce the degradation rate, enhancing the hydrogel’s stability in physiological environments [14]. In summary, microstructure (e.g., pore size, distribution, and interconnectivity) serves as the “skeleton,” directly determining mechanical strength and environmental adaptability through optimization of physical cross-linking points, molecular entanglements, and pore networks. Lack of appropriate pore structure (e.g., excessively large or uneven pores) can lead to stress concentration, accelerated degradation [35,36] and, by extension, potential loss of acupoint stimulation function—an inference drawn from the established role of mechanical integrity in device performance. Direct evidence linking microstructural parameters to therapeutic outcomes in TCVM acupoint embedding remains lacking.

### 2.3. Biocompatibility and Degradation Properties

Biocompatibility and degradation properties are core safety indicators for hydrogels as veterinary embedding materials, directly related to the safety and efficacy of their in vivo application in animals. Biocompatibility evaluation encompasses cytotoxicity, hemocompatibility, and histocompatibility. Degradation properties focus on degradation rate, degradation products, and in vivo metabolic behavior, requiring compliance with the physiological safety requirements of different animal species, ages, and application sites.

Cytotoxicity is the primary evaluation index for biocompatibility. MTT assay results demonstrate that CS/β-GP/Gel hydrogels exhibit no significant cytotoxicity toward various cell lines, including the breast cancer cell line MCF-7 and the normal breast cell line MCF-10 [14,37]. After introducing functional components, concentration must be strictly controlled to ensure low toxicity: a chitosan hydrogel loaded with 10 wt% silver titanate nanotubes (Ag-TNT) exhibits strong antibacterial activity against *Escherichia coli* (*E. coli*), while the MTT assay shows that hydrogel containing 5 wt% Ag-TNT has a half-maximal inhibitory concentration (IC_50_) of 82.87 μg/mL against cancer cells, indicating maintained low toxicity at controlled concentrations [38]; quaternized chitosan/tannic acid/gelatin (QTG) hydrogel loaded with celecoxib combines antibacterial, anti-inflammatory, and biocompatible properties [39]. Overall, chitosan/gelatin hydrogels can maintain low toxicity by optimizing polymer ratios and controlling additive concentrations.

Hemocompatibility is a key indicator for hydrogels used in acupoints rich in blood vessels (e.g., neck acupoints in animals). The hemolysis rate of chitosan/gelatin hydrogels is typically below 2%, which is well within the clinically acceptable range for blood-contacting biomaterials (ISO 10993-4:2017) and indicates good hemocompatibility [40]. In vitro hemolysis tests on chitosan/collagen/polycaprolactone hydrogel films (CSCPs) show that the hemolysis rate of all tested samples is below 1.5%, with no risk of red blood cell rupture [41]. Coagulation performance tests indicate that after mineralization modification, chitosan-based hydrogels shorten clotting time by over 41%. In a rat burn wound model, they not only rapidly stopped bleeding but also promoted wound healing with mild inflammatory response [42]. This characteristic is significant for traumatic acupoint treatment in animals.

Histocompatibility is evaluated through in vivo implantation experiments, including inflammatory response, tissue integration, and foreign body reaction. After subcutaneous implantation in mice, chitosan-based hydrogels initially induce only mild macrophage infiltration; the inflammatory response gradually subsides after 4 weeks, accompanied by ingrowth of new blood vessels [36]. This controllable inflammatory response, observed within a 4-week timeframe, indicates good short-term biocompatibility and suggests potential to promote tissue repair. The biosafety of degradation products is a key theoretical advantage for the long-term application of hydrogels: chitosan degrades into glucosamine, and gelatin degrades into small molecular peptides, both being metabolizable substances in animal bodies without known cumulative toxicity. Hyaluronic acid–chitosan-based hydrogels have been shown to completely degrade within 8 weeks in rodent models, with degradation products exhibiting antioxidant and antibacterial activities that may further support tissue repair [43]. It should be emphasized, however, that these biocompatibility and degradation data are derived primarily from short-to-medium-term subcutaneous implantation studies (up to 8 weeks) in healthy rodent models. Their extrapolation to long-term veterinary applications—particularly in chronic conditions such as canine degenerative joint disease or equine laminitis, which may require treatment durations exceeding 8 weeks—remains hypothetical at this stage and awaits systematic validation in target animal species with clinically relevant implantation periods and disease backgrounds.

Regulation of degradation properties is crucial for matching different animal tissue repair cycles, directly affecting the hydrogel’s degradation rate, drug release kinetics, cell behavior, and tissue regeneration outcomes. Research finds that in κ-carrageenan/chitosan/gelatin (KCG) composite scaffolds, adding potassium chloride (KCl) can control the degradation rate within 30% over 21 days, suitable for bone tissue repair [44]; by adjusting gelatin/casein ratio and 1-Ethyl-3-(3-dimethylaminopropyl) carbodiimide and N-Hydroxysuccinimide (EDC&NHS) concentration, the degradation cycle and drug release rate can be controlled, achieving customizable degradation properties [45]. For different application scenarios, precise regulation of degradation rate is widely employed to tailor material residence time: in bone regeneration applications, the degradation cycle is extended to over 8 weeks to match the slow process of bone repair [46]; in soft tissue repair scenarios, the degradation cycle can be flexibly regulated to 11–42 days by adjusting the chitosan concentration of thermosensitive hydrogels, so as to adapt to the relatively rapid soft tissue repair process [42]. It should be noted, however, that the degradation profiles described above are derived from non-acupoint tissue engineering and wound healing models, where the therapeutic endpoint is structural tissue regeneration rather than sustained acupoint stimulation. Their direct extrapolation to chronic TCVM applications—such as canine degenerative joint disease or equine laminitis, which require acupoint stimulation for 6–12 weeks or longer—remains a hypothesis that awaits empirical validation. Dedicated studies in target veterinary species, incorporating disease-relevant implantation periods and functional outcome measures (e.g., pain scores, joint mobility), are, therefore, necessary to establish evidence-based degradation–efficacy correlations in the context of acupoint therapy.

### 2.4. Drug-Loading Capacity and Release Properties

Drug-loading capacity and release properties are the core performance characteristics for hydrogels to achieve integrated “acupoint stimulation–drug delivery” therapy, directly determining their clinical value in animal disease treatment. Drug-loading capacity is evaluated by encapsulation efficiency and drug loading as core indicators. Release properties focus on release kinetics, triggering mechanisms, and in vivo targeted release behavior, requiring precise optimization in conjunction with the animal acupoint microenvironment, disease type, and treatment cycle.

Enhancement of drug-loading capacity relies on optimizing the interaction between the drug and the hydrogel network. For hydrophilic drugs, the porosity and cross-linking density of hydrogels are key influencing factors: the encapsulation efficiency of CS/β-GP hydrogel for levofloxacin is 65%. After introducing gelatin, due to hydrogen bond formation between gelatin amino groups and drug carboxyl groups, the encapsulation efficiency increases to 82% [47]. For hydrophobic drugs (e.g., curcumin), chitosan films load curcumin via pH responsiveness. Their amphiphilic porous structure enhances drug release under alkaline conditions, with a drug loading rate as high as 99% [48]. Nanocomposite strategies can further improve drug-loading performance: chitosan-coated poly (methacrylic acid) (PMAA) hollow spheres achieve site-specific loading of doxorubicin through electrostatic interactions, with a drug loading of 64 ± 3%. Moreover, the chitosan coating effectively inhibits drug leakage, with only 7 ± 2% leakage over 54 h in vitro [49]. This characteristic can reduce the systemic toxicity of drugs to the animal body.

Regulation of release properties is key to achieving precise treatment of animal diseases. Drug release from chitosan/gelatin hydrogels typically occurs in three stages: initial burst release, sustained release, and plateau phase. Initial burst release may cause drug concentration fluctuations and side effects, requiring technical means for suppression. Encapsulating drugs in liposomes or nanoparticles before integrating them into the hydrogel network can reduce direct surface drug exposure, effectively overcoming the burst effect: after encapsulating curcumin in oligo-conjugated linoleic acid vesicles (OCLAVs), protective sustained release is achieved via chitosan hydrogel in vitro, effectively overcoming the burst effect [50]. The sustained release phase relies on the balance between hydrogel degradation and drug diffusion. The CS/β-GP/Gel thermosensitive hydrogel system, due to the addition of gelatin, shows significantly improved mechanical strength and biocompatibility, achieving prolonged drug release. The effective drug concentration in animals is maintained longer compared to traditional formulations [13,51]. The drug release rate during the plateau phase can be accelerated by adding degradable components or nanocarriers. For example, after loading poly (betulinic acid) nanoparticles (PBA NPs) into hydroxypropyl chitosan thermosensitive hydrogel, selective drug release during the plateau phase is achieved in vitro through folate receptor targeting mechanisms, increasing drug concentration at the lesion site [52].

Drug release mechanisms and triggering conditions need to be adapted to the animal acupoint microenvironment. The drug release behavior of chitosan/gelatin hydrogels is primarily based on Fickian diffusion (Korsmeyer–Peppas model). The specific mechanism is influenced by material composition, drug properties, and environmental factors. The three-dimensional network structure of chitosan/amino acid (CS/AA) thermosensitive hydrogels (e.g., CS/Lys/β-GP, Chitosan/L-glutamic acid/β-glycerophosphate (CS/Glu/β-GP)) is stabilized by hydrogen bonds. Loading of the model drug tinidazole (TNZ) does not destroy network integrity, achieving long-term in vitro release at 37 °C [15]. Furthermore, increasing polymer concentration prolongs the release cycle. High-concentration CS/β-GP/GEL hydrogels, due to increased network density, restrict the free diffusion of drug molecules, with in vitro sustained release time exceeding 7 days [4,13]. These kinetic behaviors are attributed to the physical barrier formed by chitosan and gelatin, as well as the reversible changes in temperature-responsive cross-linking. When the environmental temperature is below the gel point, network relaxation can accelerate drug release [53,54].

The triggering mechanism of chitosan/gelatin thermosensitive hydrogels primarily relies on temperature-change-induced reorganization of the physical cross-linked network. Gelatin undergoes conformational changes at animal body temperature (37–42 °C), promoting gel network formation and slow drug release; the addition of chitosan can optimize the thermosensitive responsiveness. Adding 30% chitosan to a hydroxypropyl methylcellulose (HPMC) system reduces the gelation temperature from 66.9 °C to 43.6 °C, while adding 30% chitosan to a methylcellulose (MC) system reduces the gelation temperature from 43.6 °C to 39.3 °C, indicating that chitosan optimizes thermosensitive responsiveness by modifying polymer chain interactions [55]. This transition stems from enhanced intermolecular hydrogen bonding and hydrophobic interactions, forming a stable three-dimensional network structure at body temperature, providing a controllable release platform for drugs [15,56].

The targeting and long-lasting nature of the in vivo release behavior of chitosan/gelatin thermosensitive hydrogels have been validated through animal experiments. In a male Wistar rat brain model, a protein-loaded hydrogel achieved progressive release over 21 days, with a release rate of 43–64% in the initial 6 days and cumulative release reaching 57–83% after 1 week. Moreover, high-polymer-concentration gels showed over 4-fold volume increase within 3 weeks, with gel swelling proceeding synchronously with drug release and no burst release phenomenon [57]. This in vivo sustained-release characteristic is attributed to the physiological stability of the hydrogel, such as the cross-linked network of chitosan and gelatin resisting enzymatic hydrolysis and maintaining the gel state in response to local temperature. In a pressure ulcer model, CS/β-GP/GEL hydrogel loaded with adipose-derived mesenchymal stem cells (ADSCs) continuously released bioactive factors, promoting tissue repair without significant inflammatory response, verifying its in vivo compatibility and controllable release [13,42]. Additionally, the degradation kinetics of the hydrogel are synchronized with release; for example, in vitro studies showed that a chitosan cross-linked system completely degrades within 1–4 days, releasing 40~60% of the loaded drug, aligning with the therapeutic time window [58]. These data indicate that chitosan/gelatin hydrogels, through temperature-triggered in situ gelation, achieve localized, long-lasting drug delivery in vivo, reducing the risk of systemic exposure [4,59].

### 2.5. Multi-Stimuli Responsiveness and Synergistic Mechanism with Thermosensitivity

The synergistic effect of multi-stimuli responsiveness (e.g., pH, reactive oxygen species (ROS), enzymes) and thermosensitivity is the core mechanism for chitosan/gelatin thermosensitive hydrogels to achieve precise treatment of animal diseases. By integrating various environmental stimuli signals such as temperature, pH, ROS, and enzymes, on-demand drug release and dynamic regulation of biological functions can be achieved. This synergistic mechanism not only optimizes the intelligent performance of hydrogels but can also be extended to logic-gated release systems, further enhancing the precision, long-lasting efficacy, and safety of acupoint therapy.

The synergy between thermosensitivity and pH responsiveness is a fundamental regulatory mechanism. The gelatin component provides a body-temperature-responsive framework, achieving sol-gel transition within animal body temperature range (37–42 °C) [60,61]. Chitosan, as a pH-responsive polymer, responds to pH changes in the acupoint microenvironment through protonation/deprotonation behavior, forming electrostatic or crystalline network structures [62,63]. In low-molecular-weight chitosan hydrogels, the drug release rate reaches 95.52% within 12 h at pH 7.4, while it is only 9.82% at pH 1.2. This pH-responsive characteristic enables targeted drug release at inflammatory sites (acidic microenvironment) [64]. The synergistic effect of thermosensitivity and pH responsiveness, through dynamic covalent bonds or physical cross-linking mechanisms, not only enhances the mechanical stability of the hydrogel but also achieves “on/off” control of drug release, avoiding non-specific release [60,62].

Integration of ROS and enzyme responsiveness further enhances the precision of release regulation. In dual pH–ROS-responsive hydrogels, phenylboronic acid-modified gelatin combined with chitosan leads to boronate ester bond cleavage under high ROS levels at inflammatory sites, triggering targeted drug release. In vitro experiments show significantly increased drug release rates in the presence of ROS [65]. Similarly, enzyme responsiveness is achieved through gelatin degradation: gelatin in chitosan/gelatin systems can be gradually degraded by proteases in animals, leading to sustained release of ADSCs. In vitro wound healing experiments confirmed increased cell migration speed and a significantly increased number of ADSCs in wound tissue after 5 days [51]. Furthermore, pyrrogallol-loaded chitosan–gelatin hydrogel (Pyro-CG), under ROS response, not only enhances antibacterial activity but also achieves synergistic regulation of drug release and degradation rate [66,67].

The multi-stimuli responsive synergistic mechanism can achieve zero-order release behavior, further optimizing therapeutic effects. Hydrogels constructed via dynamic boronate ester bonds achieve constant release driven by surface erosion under pH and glucose stimulation. The cumulative drug release reaches 82.71% within 10 h (pH 7.4), significantly better than that of pure chitosan hydrogel [68,69]. This precise release characteristic can avoid drug concentration fluctuations, reducing side effects on the animal body, being especially suitable for chronic diseases requiring long-term stable drug administration.

## 3. Application of Chitosan/Gelatin Thermosensitive Hydrogels in TCVM Acupoint Catgut Embedding

### 3.1. Therapeutic Principles and Technical Bottlenecks of TCVM Acupoint Catgut Embedding

The core principle of TCVM acupoint catgut embedding is to trigger the local “de qi” effect (a sensory complex of soreness, numbness, distension, and heaviness) at the acupoint through exogenous stimuli (physical, chemical, or biological signals), thereby regulating the animal’s qi and blood circulation and visceral functions via the meridian system. While these traditional concepts are holistically defined within TCVM theory and are not fully equivalent to any single biomedical mechanism, they are partially correlated with modern observations of sensory nerve activation (for de qi), local blood flow and autonomic nervous tone (for qi and blood circulation), organ function regulation via autonomic pathways (for visceral functions), and neurovascular bundles or nerve plexuses (for meridian system). The mechanism of action involves three levels: neuro-humoral regulation, immune activation, and local microenvironment remodeling. Local sterile inflammation induced by thread implantation activates immune cells such as macrophages and lymphocytes, which release cytokines including tumor necrosis factor-α (TNF-α) and interleukin-6 (IL-6), thereby modulating the systemic immune status [70]. Simultaneously, the sustained mechanical stimulation of acupoint receptors by the thread can be transmitted to the central nervous system via spinal nerves, activating the endogenous analgesic system and promoting the release of neurotransmitters such as endorphins and enkephalins [71]. Modern research shows that acupoint catgut embedding can improve obesity-related depression by regulating the transient receptor potential vanilloid 1 (TRPV1) signaling pathway, with its mechanism related to reversing abnormal TRPV1 expression in brain regions such as the hippocampus and amygdala [70].

Although traditional acupoint catgut embedding therapy has definite efficacy in treating certain diseases, significant technical bottlenecks remain (Table 1): (1) the degradation rate of embedding materials does not match treatment needs, as catgut degrades rapidly within 2–3 weeks in animals, making it difficult to maintain long-term therapeutic effects for chronic diseases; (2) insufficient biocompatibility, with some patients experiencing redness, swelling, induration, or even rejection reactions at the embedding site; and (3) lack of drug-loading capacity, unable to achieve precise local drug delivery at the acupoint [72], making therapeutic effects reliant solely on mechanical stimulation. Although the application of biodegradable polymer threads (e.g., poly (lactic-co-glycolic acid) (PLGA), PCL) in recent years has somewhat improved biocompatibility, how to achieve “stimulation–drug delivery” integration remains unresolved. For example, a meta-analysis of obese women showed that while acupoint catgut embedding significantly reduced body mass index (BMI) and waist–hip ratio (WHR), the heterogeneity of efficacy was high (I^2^ = 92.3%), possibly related to the unstable stimulation intensity of embedding materials [73]. These limitations are similarly prevalent in TCVM clinical practice. Moreover, due to differences in animal species, body size, and anatomy, more complex requirements are placed on the material’s operability, safety, and efficacy stability.

These multifaceted limitations of traditional catgut embedding are systematically summarized and contrasted with the proposed smart hydrogel system in Table 1. This direct comparison crystallizes the core challenges in TCVM acupoint therapy and simultaneously previews the key attributes of chitosan/gelatin hydrogels that address them, namely: controllable degradation, enhanced biocompatibility, integrated drug-delivery function, and minimally invasive injectable deployment.

### 3.2. Feasibility of Chitosan/Gelatin Thermosensitive Hydrogel as a Veterinary Embedding Material

The unique physicochemical properties and biological functions of chitosan/gelatin thermosensitive hydrogels make them ideal alternative materials for TCVM acupoint catgut embedding. Their feasibility is mainly manifested in the following aspects.

Minimally Invasive Operation and In Situ Formation Adaptability: The hydrogel is in a fluid state at room temperature. After precise injection into the subcutaneous tissue of the acupoint via a syringe, it rapidly forms a three-dimensional network gel triggered by body temperature, completing the “embedding” process without surgical incision [74], significantly reducing operational difficulty and animal stress response. The chitosan/gelatin hydrogel cross-linked with β-glycerophosphate (β-GP) has a gelation time of only 63 s at 34 °C, and its osmotic pressure is close to that of animal body fluids (301.2 ± 1.5 mOsm/L), avoiding tissue irritation during injection [75]. This rapid gelation characteristic enables it to quickly fix at the target site after acupoint injection, preventing drug loss. Furthermore, through its elastic modulus, it exerts sustained mechanical traction stimulation on nerve endings around the acupoint, simulating the “retained needle” effect of traditional acupuncture.

Animal-Specific Regulation of Degradation Cycle: By adjusting chitosan deacetylation degree, gelatin content, and cross-linker concentration, the hydrogel degradation cycle can be controlled within 1–8 weeks, matching the treatment cycle needs of different animal diseases. For example, hydroxybutyl chitosan (HBC) hydrogel completely degrades after 7 weeks of subcutaneous implantation but requires 8 weeks after intramuscular implantation. This tissue-specific degradation characteristic provides a flexible time window for embedding at different acupoints [76]. For piglet viral diarrhea (treatment cycle 1–2 weeks), the formulation can be optimized to control the hydrogel degradation cycle within 10–14 days. For canine degenerative joint disease (DJD, treatment cycle 6–12 weeks), increasing cross-linking density can prolong degradation time, ensuring long-term therapeutic effects.

Excellent Veterinary Biocompatibility: Chitosan and gelatin are naturally derived polymers with well-documented biocompatibility. In 3D-printed chitosan/gelatin (CS/Gel) scaffolds, inks with a low chitosan concentration support cell attachment and proliferation, indicating favorable cytocompatibility [77]. Subcutaneous implantation in mice showed that a rapidly gelling chitosan hydrogel degraded by approximately 75% within 8 weeks. No adverse reactions were observed other than a mild local granulomatous response, suggesting acceptable safety for long-term implantation [78]. Physically blending chitosan and gelatin at a 1:1 ratio further enhanced both mechanical strength and biocompatibility, demonstrating a synergistic effect of the composite [79]. Moreover, the porous structure of the hydrogel facilitated the penetration and exchange of interstitial fluid at the acupoint, maintaining a moist microenvironment and thereby preventing local tissue ischemia caused by conventional embedding materials [80]. Separately, a polyvinyl alcohol (PVA)/chitosan/gelatin hydrogel loaded with basic fibroblast growth factor (bFGF) has demonstrated good cytocompatibility in in vivo wound repair studies, promoting fibroblast proliferation and angiogenesis [81].

Integrated “Stimulation–Drug Delivery” Function: The hydrogel can load small-molecule drugs, proteins, growth factors, or even cells through physical encapsulation, chemical bonding, or nanocomposite methods, achieving dual functions of “acupoint stimulation + drug delivery.” Chitosan/gelatin hydrogel loaded with curcumin nanoparticles achieves sustained release of curcumin for up to 28 days in glaucoma treatment, effectively reducing oxidative stress damage in trabecular meshwork cells [82]. HBC hydrogel loaded with human platelet lysate (hPL) can promote angiogenesis and collagen deposition in skin wounds through sustained release of platelet-derived growth factor (PDGF-BB) and transforming growth factor-β1 (TGF-β1) [83]. This integrated function breaks through the limitation of traditional embedding relying solely on mechanical stimulation, achieving a multiplication of therapeutic effects. Chitosan/gelatin nanocomposite hydrogel loaded with praziquantel can achieve sustained release for 10 days in vitro [84]. Levofloxacin-loaded hydrogel maintains a concentration above the minimum inhibitory concentration (MIC) in rabbit eyes after 24 h, whereas traditional eye drops fall below the MIC within 2 h [85].

### 3.3. Application Research Progress and Expansion into Veterinary Scenarios

#### 3.3.1. Research Progress on Thermosensitive Hydrogels as Integrated Platforms for Acupoint Stimulation and Drug Delivery

Thermosensitive hydrogels, by virtue of their unique “liquid injection–in situ gelation”, provide innovative material-based solutions to break through the bottlenecks in traditional acupoint catgut embedding therapy, such as uncontrollable stimulation intensity, short duration of action, and difficulty in integrating drug therapy. Existing research has confirmed the application potential of chitosan/gelatin thermosensitive hydrogels in human acupoint therapy, providing an important technical reference for TCVM applications.

Current research, through ingenious material design and delivery strategy innovation, has successfully constructed various acupoint therapy platforms based on thermosensitive hydrogels. The core lies in achieving the integration and synergy of “needle” and “drug.” Among them, the most representative strategy is developing drug-loaded modified acupuncture needles. Liu et al. (2024) designed a hollow acupuncture needle (HC-EA) with an etched honeycomb-like structure at its tip, and its high-loading cavity can be filled with melittin-loaded thermosensitive hydrogel (MLT-Gel) [86]. This hydrogel uses chitosan (CS)/β-glycerophosphate (β-GP)/hyaluronic acid (HA) as the matrix and possesses acidic microenvironment-responsive properties. In a rheumatoid arthritis (RA) mouse model, this “needle–gel” composite, while performing electroacupuncture stimulation, intelligently releases the drug in the acidic environment of the inflammatory joint, significantly restoring Th17/Treg immune balance and reducing levels of inflammatory factors like TNF-α, IL-6, and IL-1β at the lesion site [86]. Another study on RA employed a strategy of acupoint injection of a nanocomposite hydrogel, with its unique advantage lying in revealing the phenomenon of “acupoint-targeted accumulation.” Ren et al. (2021) found that 48 h after acupoint injection of a composite hydrogel loaded with triptolide nanoparticles and the adenosine receptor agonist CCPA, the drug accumulation in arthritic paws was 13.5% higher than that in the non-acupoint injection group [8]. This pharmacokinetic characteristic directly explains the hydrogel’s enhanced efficacy and reduced systemic toxicity. Simultaneously, the analgesic effect produced by CCPA locally at the acupoint (mechanical pain threshold increase 4.9 times that of non-acupoints) also highlights the site-specific advantage of acupoint drug delivery [8].

Besides combination with acupuncture needles, thermosensitive hydrogels as independent acupoint injection formulations also show broad prospects in pain and neurological disease management. Wei et al. (2022) prepared a CS/β-GP thermosensitive hydrogel loaded with dexamethasone and Fe_3_O_4_ nanoparticles (the CS/GP/Fe_3_O_4_/DXM hydrogel) for acupoint embedding in sciatica rats [87]. This system not only provides sustained local anti-inflammatory drug release, but its magnetic component also allows remote physical stimulation of the acupoint under an applied external magnetic field, thereby exerting analgesic effects through multiple mechanisms, including inhibiting the p38MAPK phosphorylation pathway. This marks the birth of a new mode of “physical–chemical combined” acupoint stimulation [87]. In more complex neurological diseases such as Parkinson’s disease (PD), Chen et al. (2025) developed an injectable, conductive, self-healing hydrogel based on chitosan (chitosan/polydopamine-coated polyurethane (CPUD)) and applied it in combination with acupuncture in a PD rat model [88]. The combination therapy not only significantly promoted dopaminergic neuron regeneration (>80%) but, more crucially, achieved precise regulation of neuroinflammation by polarizing microglia from the pro-inflammatory M1 phenotype to the reparative M2 phenotype (M2/M1 ratio increased by about 12.6 times), thereby synergistically improving the animals’ motor function [88]. These studies confirm that chitosan/gelatin thermosensitive hydrogels, through the synergistic effect of “physical stimulation–drug delivery,” have significant advantages in scenarios such as pain management and neural repair. Their core technology can be transferred to TCVM clinical applications.

#### 3.3.2. Specific Advantages and Functional Expansion Applications of Chitosan/Gelatin Thermosensitive Hydrogels

Among various hydrogel systems suitable for TCVM acupoint embedding, chitosan/gelatin-based thermosensitive hydrogels occupy a central position in the aforementioned application explorations due to their natural material source, excellent biocompatibility, flexibly adjustable physicochemical properties, and inherent bioactivity, highlighting irreplaceable application value and providing strong support for TCVM clinical translation. On one hand, this system is a high-quality carrier for building intelligent responsive drug delivery systems. The acidic-responsive drug release characteristic of the CS/β-GP/HA hydrogel used by Liu et al. (2024) for RA treatment precisely relies on the inherent property of chitosan’s increased solubility in acidic environments, accurately matching the inflammatory lesion microenvironment to achieve targeted drug release therapy for the diseased site [86]. This responsive design targeting the local microenvironment of lesions (e.g., pH value, specific enzyme expression) is the core mechanism for chitosan-based hydrogels to achieve precise drug delivery and on-demand release, effectively improving drug utilization in veterinary clinical practice and reducing systemic toxicity. On the other hand, the good cell affinity and modifiability of this system make it an excellent carrier for loading biologically active components such as cells and growth factors. In a study on diabetic skin wound healing, Chen et al. (2021) combined a hydrogel/cryogel dressing loaded with adipose-derived stem cells with acupuncture at acupoints around the wound [89]. This combined regimen enhanced the synergistic effect of local immune microenvironment regulation by upregulating reparative cytokines like stromal cell-derived factor-1 (SDF-1) and transforming growth factor-β1 (TGFβ-1) while downregulating pro-inflammatory factors like TNF-α and IL-1β, achieving a wound closure rate exceeding 90% within 8 days [89]. This result confirms that chitosan/gelatin hydrogel can not only deliver small-molecule drugs but also serve as an “active cell carrier” and “growth factor sustained-release reservoir,” forming deep synergy with acupoint stimulation and providing new pathways for treating complex diseases in TCVM.

Furthermore, by compounding functional components such as conductive materials and nanoparticles, the functional boundaries of chitosan/gelatin hydrogels have been significantly broadened. Zhang et al. (2025), in spinal cord injury treatment, implanted a conductive gelatin hydrogel compounded with carbon nanotubes into the injury site, combined with electroacupuncture stimulation at Huatuo Jiaji acupoints [90]. This “material conductivity + acupoint electroacupuncture” dual electrical synergy strategy, both internal and external, effectively reconstructs the neural electrical microenvironment in the injured area, not only promoting nerve axon regeneration but also inhibiting inflammatory response and glial scar formation by downregulating the janus kinase–signal transducer and activator of transcription (JAK-STAT) signaling pathway, ultimately synergistically improving motor and bladder functions [90]. This marks that such hydrogels have evolved into multifunctional biomaterials capable of intervening in neuro-electrophysiological regulation and aiding complex tissue regeneration, providing innovative directions for treating neurological injury diseases in TCVM.

#### 3.3.3. Core Advantages for Expansion into the TCVM Field

The core advantages of expanding chitosan/gelatin thermosensitive hydrogels into the TCVM field lie in the universality and tunability of their properties, enabling precise matching with the special needs of animal disease treatment:

Cross-Species Adaptability: The gelation temperature, degradation rate, and mechanical properties of the hydrogel can be adapted across species through formulation adjustments. It can meet the body temperature requirements of homeothermic animals such as pigs and dogs (37.5–40 °C) and can also be adapted to the body temperature environment of poultry such as chickens and ducks (40–42 °C) via optimized formulations. The anatomical differences in acupoints in different animals [91,92] (e.g., subcutaneous tissue thickness, blood vessel and nerve distribution) can be precisely matched by adjusting the hydrogel’s mechanical modulus and injection formulation properties (e.g., viscosity, needle compatibility).

High Treatment Safety: The biocompatibility and degradability of natural polymer-based materials ensure the application safety of the hydrogel in animals. The degradation products have no cumulative toxicity, avoiding potential long-term side effects from synthetic chemical materials [93]. The local drug delivery characteristic of the hydrogel reduces drug damage to animal organs like the liver and kidneys, being especially suitable for young and elderly animals with weaker liver and kidney function.

Strong Operational Convenience: The injectability of the hydrogel makes it compatible with acupoint injection operations in TCVM clinical practice. It does not require complex surgical equipment; the embedding process can be completed using conventional syringes, reducing the technical requirements for operators. Simultaneously, it avoids the stress response caused by repeated capture and surgical trauma, significantly improving animal welfare and facilitating promotion and application in primary veterinary institutions [94].

Good Cost Controllability: Both chitosan and gelatin are natural materials with wide sources and a low cost [95,96]. Their scaled-up production processes are mature. Compared to synthetic polymer materials like PLGA and PCL, they significantly reduce the production cost of veterinary formulations, meeting the economic needs of animal husbandry and companion animal medicine.

Outstanding Green Adaptability: This hydrogel highly aligns with the industrial needs of green farming and sustainable animal husbandry development. Its base materials are natural renewable biopolymers. Chitosan, in particular, is a high-value utilization product of aquaculture by-products. Its production process is low-energy and low-pollution, aligning with circular economy concepts. The characteristic of local precise sustained release at acupoints can significantly reduce the systemic drug dosage. Combined with chitosan’s natural antibacterial activity, it can partially replace antibiotic use, addressing the pain point of antibiotic abuse in farming [97]. Its biodegradability allows it to be metabolized into non-toxic small molecules in vivo, without accumulating or causing secondary pollution to the farming environment, aiding the ecological farming model combining planting and breeding. Meanwhile, its integrated treatment function can reduce the stress stimulation of repeated drug administration on animals, improving disease cure rates and animal production performance, considering both animal welfare and farming economic benefits. Furthermore, empowering traditional TCVM therapies with modern material technology enables the application of traditional green diagnostic and therapeutic wisdom in large-scale livestock farming, which may act as a potential bridge between TCVM and the sustainable development of animal husbandry.

#### 3.3.4. Application Explorations for Specific TCVM Diseases

Deeply integrating chitosan/gelatin thermosensitive hydrogels with veterinary acupuncture technology and further expanding into the prevention and treatment of diseases in economic and companion animals also have clear application prospects and huge translational potential [98]. The following disease-specific scenarios are proposed as illustrative and forward-looking concepts, grounded in the physicochemical and biological properties of chitosan/gelatin thermosensitive hydrogels discussed in Section 2 and Section 3.2, as well as the pathophysiological characteristics of each condition. These formulations and therapeutic strategies have not yet been clinically validated in veterinary acupoint therapy; they are intended to stimulate further research and to provide a conceptual framework for future TCVM biomaterial development.

Piglet diarrhea is a common infectious disease in the pig industry. Traditional treatment methods suffer from low drug utilization and slow intestinal mucosal repair. For this disease, a chitosan/gelatin thermosensitive hydrogel loaded with effective natural product molecules (e.g., astragalus polysaccharides, berberine) or intestinal mucosal protective agents (e.g., montmorillonite) can be designed for Houhai acupoint injection administration in piglets [99,100,101]. The hydrogel forms persistent physical stimulation at the acupoint, regulating intestinal-related neuro-immune pathways, while continuously releasing drugs directly targeting the local intestinal area, potentially achieving a synergistic effect of “acupoint regulation + drug therapy.” This proposed strategy, while supported by the known pharmacological activities of astragalus polysaccharides and berberine, awaits experimental validation in porcine acupoint models.

Canine DJD is a common chronic disease in companion animals, manifested as joint pain and limited mobility. Traditional treatment relies on long-term oral non-steroidal anti-inflammatory drugs (NSAIDs), carrying risks of gastrointestinal side effects [102]. Developing a chitosan/gelatin thermosensitive hydrogel loaded with NSAIDs (e.g., meloxicam) or cartilage repair factors (e.g., chondroitin sulfate) for embedding at canine acupoints like “Housanli” (ST36) or “Yanglingquan” (GB34) can provide long-lasting acupoint stimulation to relieve pain while maintaining long-term drug concentration locally in the joint [103,104]. This approach can reduce systemic drug dosage and frequency, improving treatment safety and compliance. This illustrative paradigm, combining sustained acupoint stimulation with intra-articular drug targeting, is derived from the mechanical tunability and drug-loading capacity of the hydrogel [102,103]; its clinical feasibility in canine patients remains to be investigated.

In animal wound treatment, chitosan/gelatin thermosensitive hydrogel loaded with growth factors (e.g., basic fibroblast growth factor (bFGF)) or antibacterial drugs (e.g., levofloxacin) can be injected into acupoints around the wound. Through acupoint stimulation, it regulates local blood circulation and immune function while continuously releasing drugs to promote wound healing and prevent infection [81,105,106]. Research by Chen et al. (2021) showed that combining a chitosan-based hydrogel loaded with ADSCs with acupuncture around the wound upregulated reparative cytokines like SDF-1 and TGFβ-1 while downregulating pro-inflammatory factors like TNF-α and IL-1β, achieving over 90% wound closure within 8 days [89]. This result provides an important reference for acupoint treatment of animal wounds. It should be emphasized, however, that this combined approach has only been demonstrated in rodent models; its translation to veterinary wound care, including acupoint selection and hydrogel formulation optimization, represents a future research direction rather than an established protocol.

Bovine and equine laminitis are characterized by hoof pain, lameness, and abnormal hoof horn metabolism. Traditional treatment relies on analgesic and anti-inflammatory drugs and hoof care, suffering from short-lived efficacy and recurrent episodes. Clinically, acupuncture at acupoints like “Qiantimen” and “Baihui” (GV20) is often used to regulate qi and blood and relieve pain [107]. For this disease, a chitosan/gelatin thermosensitive hydrogel loaded with NSAIDs (e.g., flunixin meglumine) or mesenchymal stem cells can be designed for embedding at hoof acupoints. The hydrogel regulates hoof microcirculation through persistent acupoint stimulation while slowly releasing drugs targeting the inflammatory site, reducing pain and promoting hoof horn repair [108,109]. This approach can extend drug efficacy duration to 7–10 days, reducing administration frequency and minimizing gastrointestinal damage from long-term drug use in cattle. This conceptual framework, while rational based on the anti-inflammatory and pro-reparative properties of flunixin meglumine and MSCs [107,108], requires rigorous efficacy and safety assessment in target species under field conditions.

Horses, as working or sport animals, have a high incidence of thoracolumbar myofascitis, manifested as back muscle spasms, stiffness, and decreased performance. Traditional treatments include acupuncture at acupoints like “Baihui” (GV20), “Shenshu” (BL23), and “Ashi” points, electroacupuncture stimulation, and muscle relaxants. However, muscle relaxants have a short duration of action and require repeated administration [110,111]. Developing a chitosan/gelatin thermosensitive hydrogel loaded with muscle relaxants (e.g., methocarbamol) and anti-inflammatory factors (e.g., IL-1 receptor antagonist) for embedding at relevant back acupoints in horses can simulate continuous acupuncture effects through the elastic mechanical stimulation of the hydrogel, relieving muscle spasms, while slowly releasing drugs to suppress local inflammation and improve muscle metabolism. This approach adapts to the anatomical properties of equine back acupoints, maintaining therapeutic effects for 1–2 weeks and reducing the frequency of human intervention, aiding sport horses in quickly recovering their athletic ability. Moreover, the hydrogel has excellent biocompatibility and will not induce tissue rejection reactions in the horse’s back area. As with the other scenarios outlined in this section, this application is proposed as a forward-looking hypothesis, informed by the material’s biocompatibility profile and the clinical presentation of equine myofasciitis; dedicated equine studies are needed to confirm its therapeutic value and local tissue compatibility.

To provide a clear overview of these tailored strategies, the formulation adjustments, target acupoints, and therapeutic mechanisms for the major TCVM diseases discussed above are synthesized in Table 2. This summary underscores the versatility and clinically driven design of chitosan/gelatin thermosensitive hydrogels for modernizing acupoint embedding therapy across a spectrum of veterinary conditions.

## 4. Summary and Outlook

### 4.1. Main Conclusions

Chitosan/gelatin thermosensitive hydrogels represent a class of intelligent biomaterials whose core advantages stem from the natural synergy between chitosan and gelatin and the precise tunability of their thermosensitive responsiveness. The key conclusions of this review can be summarized as follows:

- Multifunctional performance platform: These hydrogels integrate injectability, body-temperature-triggered in situ gelation, on-demand mechanical tunability, excellent biocompatibility, programmable degradation (1–8 weeks), high drug-loading capacity, and multi-stimuli responsiveness (temperature/pH/ROS/enzyme), collectively forming an integrated therapeutic platform combining physical stimulation, chemical delivery, and biological regulation.
- Overcoming traditional TCVM bottlenecks: In acupoint embedding applications, this material addresses the long-standing limitations of traditional catgut—including unstable degradation, insufficient biocompatibility, and lack of drug-loading capacity—while offering minimally invasive injection and sustained acupoint stimulation through its elastic mechanical properties.
- Cross-species adaptability and application potential: The tunable gelation temperature (37–42 °C) and degradation kinetics allow adaptation to diverse animal species (e.g., pigs, dogs, horses, poultry). Prospective applications in diseases such as piglet diarrhea, canine degenerative joint disease, and equine laminitis illustrate the potential of an integrated “stimulation–drug delivery” strategy.

Collectively, this innovative biomaterial platform provides a technological pathway for modernizing TCVM acupoint therapy—addressing issues of experience-dependent operation, inconsistent efficacy, and low delivery efficiency—while showcasing broad interdisciplinary translational potential at the intersection of traditional medicine and advanced materials science.

### 4.2. Current Challenges and Limitations

**Figure 4:**
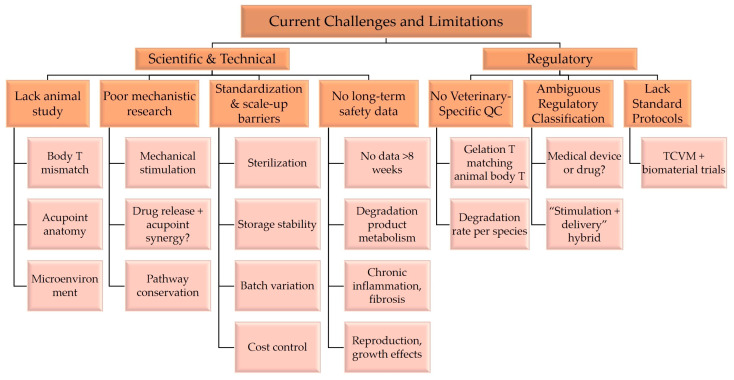
Current translational challenges in TCVM: scientific/technical and regulatory aspects.

Although research on chitosan/gelatin thermosensitive hydrogels in human medicine has made significant progress, their translation to the TCVM field still faces multiple targeted challenges (Figure 4).

Lack of Animal-Specific Research: The normal body temperatures of different livestock species (e.g., dogs, pigs, cattle) are significantly higher than those of humans and rodent models. Existing gelation temperature formulas designed for human body temperature cannot be directly adapted. There are significant differences in acupoint anatomical localization among different species and breeds. There is a lack of systematic basic data on subcutaneous tissue thickness, fascial layers, local vascular/nerve distribution, and microenvironment properties (e.g., pH value, enzyme profile composition) that would support the precise regulation of hydrogel injection depth, gel morphology, and interaction with acupoint receptors, directly affecting stimulation efficiency and therapeutic efficacy stability [112].

Insufficient Depth of Mechanistic Research: Existing related explorations mostly remain at the level of efficacy observation in animal models, lacking sufficient elucidation of the interaction mechanism among “hydrogel–acupoint–animal body,” especially a multi-level analysis from molecular–cellular–whole organism perspectives. For example, how the mechanical stimulation of the hydrogel activates the animal’s neuro-humoral regulatory network through acupoint receptors, how drug sustained release and acupoint stimulation synergistically regulate immune cell polarization and cytokine secretion, and the conservation and specificity of related signaling pathways (e.g., TRPV1, p38MAPK) in different animal species are not yet clear, limiting the precise optimization of treatment protocols.

Difficulties in Formulation Standardization and Scale-up: TCVM clinical application has stringent requirements for formulation stability, safety, and cost control, but currently lacks standardized preparation processes adapted to veterinary scenarios. During the sterilization process, high-temperature steam sterilization can easily damage the thermosensitive cross-linked structure of hydrogels, whereas irradiation sterilization may induce polymer chain degradation. It is necessary to develop mild and efficient sterilization technologies (such as filtration sterilization combined with aseptic filling) [113,114]. During storage, the stability of the hydrogel sol state, shelf life, and performance degradation patterns under low/high-temperature conditions are not yet clear, limiting its feasibility for field application. For large-scale production, there is a lack of mature technical solutions to support batch-to-batch variations in natural polymer raw materials, precise control of cross-linker dosage, and production cost optimization [35,115].

Lack of Long-Term Safety Evaluation: Existing research mostly focuses on short-term (≤8 weeks) degradation behavior and biocompatibility [78]. However, some TCVM diseases (e.g., Bi syndrome, intervertebral disc disease) require long-term treatment. Safety data for over 6 months in vivo are severely lacking. The metabolic pathways of long-term degradation products in animals, potential organ accumulation effects, whether long-term implantation induces chronic inflammation, tissue fibrosis, or immune dysfunction, and potential impacts on animal reproductive performance and growth development all lack systematic experimental validation, becoming major obstacles to clinical translation.

### 4.3. Regulatory Challenges in TCVM Clinical Translation

As a cross-disciplinary product combining smart biomaterials and Traditional Chinese Veterinary Medicine, chitosan/gelatin thermosensitive hydrogels face unique regulatory challenges in veterinary clinical licensing and certification. Currently, the regulatory framework for TCVM-related biomaterials is still in the exploratory stage in most countries, with no unified industry standards for the formulation, quality control, and efficacy evaluation of acupoint embedding materials. For chitosan/gelatin hydrogels, the key regulatory difficulties include: (1) the lack of veterinary-specific quality evaluation indicators for thermosensitive hydrogels (e.g., gelation temperature matching animal body temperature, degradation rate adapted to different animal species); (2) the difficulty in defining the regulatory classification of “acupoint stimulation + drug delivery” integrated materials (as veterinary medical devices or veterinary drugs); and (3) the lack of standardized clinical trial protocols for TCVM combined with biomaterials. In China, the regulatory approval of such materials needs to comply with the Veterinary Drug Administration Law and Regulations on the Supervision and Administration of Medical Devices for Animals, with additional requirements for the validation of TCVM theoretical compatibility. However, the current regulatory flexibility for traditional veterinary therapy combined with innovative biomaterials provides opportunities for the approval of chitosan/gelatin hydrogels—especially for green and antibiotic-free formulations that align with the development of sustainable animal husbandry.

### 4.4. Future Research Directions

**Figure 5:**
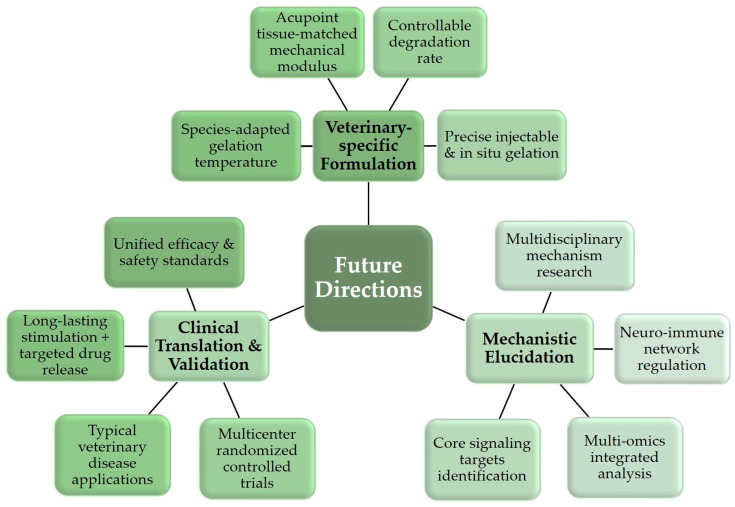
Future research directions for chitosan/gelatin hydrogels in TCVM.

Addressing the above challenges and combining the development trends of materials science and TCVM, future research should focus on the following directions to promote the standardized application of chitosan/gelatin thermosensitive hydrogels in the TCVM field (Figure 5).

Develop Veterinary-Specific Formulation Systems: Precisely optimize formulations based on the physiological properties of different livestock species. Targeting the body temperature differences in species like dogs, pigs, cattle, and horses, regulate the gelation temperature to 39–41 °C by adjusting chitosan deacetylation degree, gelatin molecular weight, and β-glycerophosphate concentration, ensuring injectability and rapid in vivo gelation stability. Combine imaging techniques to systematically characterize the subcutaneous tissue thickness and vascular/nerve distribution of target acupoints, optimizing the hydrogel’s mechanical modulus (e.g., for canine acupoints around joints, increase compressive modulus to 10~20 kPa to maintain long-lasting mechanical stimulation; for pig Houhai acupoints, adapt to loose subcutaneous tissue by selecting low-modulus formulas to avoid tissue damage). Based on the local enzyme profile properties of different animals, introduce appropriate amounts of hyaluronic acid or cross-linkers to adjust the degradation cycle, precisely matching the hydrogel degradation rate with the animal disease treatment cycle.

Deepen Mechanistic Exploration and Technological Innovation: Utilize multidisciplinary technical means to dissect the mechanisms of action. Use gene knockout animal models to identify key signaling molecules for acupoint stimulation. Monitor real-time changes in the electrical activity of acupoint nerve endings under hydrogel stimulation using fiber photometry recording technology. Combine transcriptomics, proteomics, and metabolomics analysis to systematically elucidate the molecular pathways by which hydrogel mechanical stimulation and drug sustained release synergistically regulate the animal’s neuro-immune network, identifying core regulatory targets for macrophage polarization, cytokine secretion, and neurotransmitter release, providing a theoretical basis for treatment protocol optimization.

Accelerate Clinical Translation and Evidence-Based Medicine Validation: Design multicenter, large-sample, randomized controlled trials (RCTs) oriented towards TCVM clinical needs, constructing a scientifically rigorous evidence-based evaluation system to provide high-level evidence support for the clinical promotion of hydrogels. For canine degenerative osteoarthritis, a common chronic disease in companion animals, classic acupoints for relieving pain and promoting blood circulation such as Housanli, Yanglingquan, and Xuanzhong should be selected. Inject chitosan/gelatin thermosensitive hydrogel loaded with NSAIDs (meloxicam) and cartilage repair factors (chondroitin sulfate). Set up three control groups: a traditional catgut embedding group, a simple drug acupoint injection group, and a blank hydrogel group. Use core quantitative indicators such as Glasgow pain score, joint range of motion, serum TNF-α/IL-6 levels, and changes in bone density on joint imaging. Track continuously for 12 weeks to systematically verify the synergistic clinical advantage of the hydrogel’s “long-lasting stimulation + targeted drug release,” clarifying the extent of efficacy improvement and safety differences compared to traditional therapies. For bovine mastitis (a representative disease with a high incidence in animal husbandry clinics and effective intervention by acupuncture), focus on acupuncture commonly used acupoints like Rugen (ST18), Danzhong (CV17), and Lingtai (GV10). Apply hydrogel loaded with antimicrobial peptides and anti-inflammatory factors for acupoint embedding, optimizing the hydrogel’s mechanical modulus to adapt to the anatomical properties of mammary region acupoints (avoiding compression of mammary tissue). Set an acupuncture treatment group and conventional antibiotic injection group as controls. Evaluate the application value of the hydrogel in reducing antibiotic abuse, extending treatment duration, and reducing the recurrence rate by monitoring milk somatic cell count, udder swelling degree, inflammatory factor content in milk, and bacterial clearance rate. Simultaneously record changes in milk yield, considering both efficacy and farming economic benefits. Synchronously establish unified efficacy evaluation standards for TCVM acupoint hydrogel therapy, clarify adverse reaction grading (e.g., criteria for determining local redness/swelling, rejection reactions) and monitoring procedures, standardize sample size estimation, blinding design, and data statistical methods, forming a replicable clinical validation protocol, laying the foundation for incorporating hydrogel therapy into TCVM clinical diagnosis and treatment guidelines.

**Figure 6:**
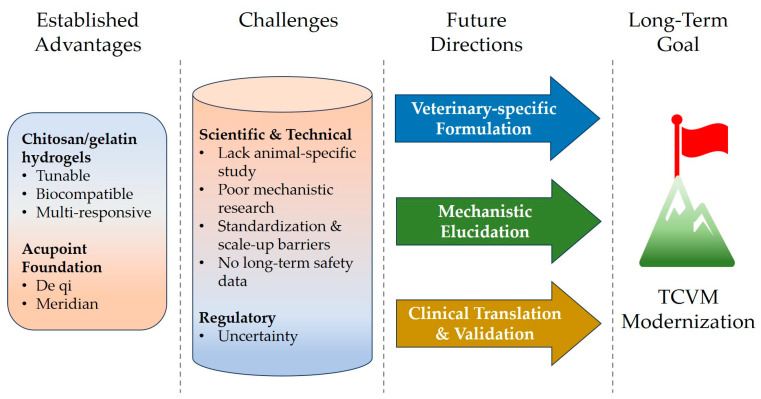
From foundation to future: a conceptual roadmap of chitosan/gelatin hydrogels for TCVM translation.

Relying on the full-chain strategy encompassing veterinary formulation customization, mechanism elucidation, clinical translation, and intelligent upgrades, chitosan/gelatin thermosensitive hydrogels will achieve a functional leap in the TCVM field, providing a feasible technological pathway for the standardization and modernization of traditional acupuncture therapy. This innovative model of “traditional therapy + modern materials” can not only enhance the precision and safety of animal disease diagnosis and treatment but also strengthen the core competitiveness of TCVM. In the future, it will be necessary to accelerate technology transfer and standard system development guided by veterinary clinical needs. As summarized in Figure 6, this integrated effort—spanning material advantages, TCVM theory, barrier resolution, and targeted research—aims to facilitate the translation of this technology from the laboratory to veterinary practice, thereby contributing to green animal healthcare and the sustainable development of the livestock industry.

## Figures and Tables

**Table 1:** Comparative analysis of traditional acupoint embedding materials and chitosan/gelatin thermosensitive smart hydrogels.

Performance Parameter	Traditional Embedding Materials	Chitosan/Gelatin Smart Hydrogels	Implications for TCVM
Degradation Controllability	Unstable; usually degrading within 2–3 weeks *	Tunable period *	Synchronizes treatment cycle with material degradation
Biocompatibility	May cause redness, swelling, induration, rejection *	Excellent; safe degradation products *	Reduces adverse reactions
Drug-loading Capacity	None (mechanical-only stimulation) *	High capacity for drugs/cells *	Enables “stimulation + delivery”
Stimulation Controllability	Uncontrollable intensity; high variability *	Controllable via mechanics and release ^#^	Improves efficacy consistency
Operational Convenience	Invasive implantation (puncture/incision) *	Minimally invasive injection (sol-gel) *	Reduces stress and complexity
Environmental Responsiveness	None *	Multi-stimuli (temp, pH, ROS, enzyme) *	Enables precise, on-demand therapy

* Reported in cited literature. ^#^ Inferred from literature-based physicochemical or biological data; direct validation in TCVM acupoint settings is not yet available.

**Table 2:** Proposed formulation strategies and illustrative application paradigms of chitosan/gelatin thermosensitive hydrogels for potential TCVM diseases interventions.

Disease.	Target Acupoints	Projected Treatment Cycle	Key Properties Optimization	Loaded Agents (Examples)	Proposed Therapeutic Advantage
Piglet Diarrhea	CV-1	1–2 wks ^#^	Fast gelation; 10–14 day degradation *	APS, berberine, montmorillonite ^#^	Gut immunomodulation + local drug synergy ^§^
Canine DJD	ST36, GB34	6–12 wks ^#^	High modulus, prolonged degradation *	Meloxicam, chondroitin sulfate, MSCs ^#^	Long-term pain relief and cartilage repair ^§^
Wound Healing	Periwound	2–4 wks ^#^	Pro-angiogenic pores; moderate degradation *	bFGF, Levofloxacin, ADSCs ^#^	Accelerating healing and preventing infection ^§^
Laminitis	Hoof acupoints	4–8 wks ^#^	Hoof-adapted mechanics; ~6 wk degradation ^#^	Flunixin meglumine, MSCs ^#^	Sustained anti-inflammatory and analgesia ^§^
Equine Thoracolumbar Myofasciitis	GV20, (BL23)	1–2 wks ^#^	Muscle-adaptable modulus; 1–2 wk degradation ^#^	Methocarbamol, IL-1Ra ^#^	Muscle relaxation and anti-inflammation ^§^

* Reported in cited literature. ^#^ Inferred from literature-based physicochemical or biological data; direct validation in TCVM acupoint settings is not yet available. ^§^ Proposed as a forward-looking, illustrative concept in this review.

## Data Availability

Not applicable.

## Abbreviations

ADSCs	Adipose-derived Mesenchymal Stem Cells
Ag-TNT	Silver Titanate Nanotubes
APS	Astragalus Polysaccharides
bFGF	Basic Fibroblast Growth Factor
CB-nHA	Cuttlebone-derived Nanohydroxyapatite
CMC	Carboxymethyl Chitosan
CPUD	Chitosan/Polydopamine-Coated Polyurethane
CS	Chitosan
CS/AA	Chitosan/Amino Acid
CS/β-GP	Chitosan/β-glycerophosphate
CS/β-GP/Gel	Chitosan/β-glycerophosphate/gelatin
CS/Gel	Chitosan/gelatin
CS/Glu/β-GP	Chitosan/L-glutamic acid/β-glycerophosphate
CS/Lys/β-GP	Chitosan/L-lysine/β-glycerophosphate
CSCPs	Chitosan/Collagen/Polycaprolactone Hydrogel Films
CV17	Danzhong Acupoint (Conception Vessel 17)
DJD	Degenerative Joint Disease
DXM	Dexamethasone
EDC&NHS	1-Ethyl-3-(3-dimethylaminopropyl) carbodiimide and N-Hydroxysuccinimide
G′	Storage Modulus
G″	Loss Modulus
Gel	Gelatin
GV-1	Houhai Acupoint (Conception Vessel 1)
GV10	Lingtai Acupoint (Governor Vessel 10)
GV20	Baihui Acupoint (Governor Vessel 20)
HA	Hyaluronic Acid
HBC	Hydroxybutyl Chitosan
HC-EA	Hollow Acupuncture Needle
HPMC	Hydroxypropyl Methylcellulose
hPL	Human Platelet Lysate
IC50	Half-Maximal Inhibitory Concentration
IL-1β	Interleukin-1β
IL-1Ra	Interleukin-1 Receptor Antagonist
IL-6	Interleukin-6
JAK-STAT	Janus Kinase–Signal Transducer and Activator of Transcription
KCG	κ-carrageenan/chitosan/gelatin
KCl	Potassium Chloride
LAP	Laponite
MIC	Minimum Inhibitory Concentration
MC	Methylcellulose
MSCs	Mesenchymal Stem Cells
MLT-Gel	Melittin-loaded Thermosensitive Hydrogel
MRI	Magnetic Resonance Imaging
NSAIDs	Non-Steroidal Anti-Inflammatory Drugs
OCLAVs	Oligo-conjugated Linoleic Acid Vesicles
PBA NPs	Poly (betulinic acid) Nanoparticles
PD	Parkinson’s Disease
PDGF-BB	Platelet-Derived Growth Factor-BB
PMAA	Poly (methacrylic acid)
PCL	Polycaprolactone
PLGA	Poly (lactic-co-glycolic acid)
p38MAPK	p38 Mitogen-Activated Protein Kinase
Pyro-CG	Pyrrogallol-loaded Chitosan-Gelatin Hydrogel
QCS	Quaternized Chitosan
QTG	Quaternized chitosan/tannic acid/gelatin
RA	Rheumatoid Arthritis
ROS	Reactive Oxygen Species
RCTs	Randomized Controlled Trials
SDF-1	Stromal Cell-Derived Factor-1
ST18	Rugen Acupoint (Stomach Meridian 18)
ST36	Housanli Acupoint (Stomach Meridian 36)
TGF-β1	Transforming Growth Factor-β1
TNF-α	Tumor Necrosis Factor-α
TNZ	Tinidazole
TCVM	Traditional Chinese Veterinary Medicine
TRPV1	Transient Receptor Potential Vanilloid 1
WHR	Waist–Hip Ratio
BMI	Body Mass Index
BL23	Shenshu Acupoint (Bladder Meridian 23)
GB34	Yanglingquan Acupoint (Gallbladder Meridian 34)
